# Development of a Rapid and Sensitive AlphaLISA-Based Assay for Lassa Virus Glycoprotein Detection

**DOI:** 10.3390/pathogens15030243

**Published:** 2026-02-25

**Authors:** Hao Cai, Qingyu Lv, Wenhua Huang, Shaolong Chen, Peng Liu, Hua Jiang, Qian Li, Decong Kong, Yuhao Ren, Zhongpeng Zhao, Chengsong Wan, Yongqiang Jiang

**Affiliations:** 1 School of Public Health, Southern Medical University, Guangzhou 510515, China; caihao24601@163.com; 2 State Key Laboratory of Pathogen and Biosecurity, Academy of Military Medical Sciences, Beijing 100071, China; lvqingyu2004@126.com (Q.L.); huangwh1993@163.com (W.H.); kantchen@163.com (S.C.); ammsliupeng@163.com (P.L.); jhua76@126.com (H.J.); liqian_bime@163.com (Q.L.); kongdecong-118@163.com (D.K.); www2354@126.com (Y.R.); xiaozzp@163.com (Z.Z.)

**Keywords:** Lassa virus, single B-cell antibody technology, rabbit monoclonal antibody, AlphaLISA, viral detection

## Abstract

Lassa virus (LASV), a member of the Arenaviridae family, is the causative agent of Lassa fever (LF), an acute zoonotic hemorrhagic disease transmitted by rodents, characterized by high infectivity and mortality rates. Due to the nonspecific nature of early clinical symptoms, the development of rapid, sensitive, and specific diagnostic methods is critical for effective epidemic control. In this study, the Lassa virus glycoprotein complex (LASV-G) was selected as the target antigen. High-affinity rabbit monoclonal antibodies were generated using a single B-cell cloning approach, and an AlphaLISA (Amplified Luminescent Proximity Homogeneous Assay)-based homogeneous, no-wash detection system was established. Sixteen LASV-G-specific monoclonal antibodies were isolated through flow cytometric sorting, and the optimal antibody pair (56–24) was identified by AlphaLISA pairing and performance screening. The established AlphaLISA system exhibited a limit of detection (LOD) of 0.025 ng/mL, representing approximately a 30-fold increase in sensitivity compared with conventional Enzyme Linked Immunosorbent Assay (ELISA), while reducing the total assay time to less than 30 min. The coefficient of variation (CV) was below 8%, and no cross-reactivity was observed with Ebola, dengue, yellow fever, Zika, or influenza virus antigens. These findings demonstrate that the developed AlphaLISA assay possesses high sensitivity, rapid detection, and good tolerance to matrix effects, significantly improving the efficiency of early LASV antigen detection. This work provides a potential platform for the rapid on-site screening and epidemiological surveillance of highly pathogenic viruses.

## 1. Introduction

Lassa virus (LASV), a member of the Arenaviridae family, is the causative agent of Lassa fever (LF). This rodent-borne zoonotic hemorrhagic disease is widely endemic in West Africa, causing an estimated 100,000 to 300,000 infections and approximately 5000 deaths annually, based on prospective epidemiological studies conducted in Sierra Leone [1]. Current outbreaks are mainly concentrated in Nigeria, Sierra Leone, Liberia, and Guinea [2,3]. The multimammate rat (*Mastomys natalensis*) is recognized as the natural reservoir of LASV, from which the virus was first isolated during outbreaks in Sierra Leone [4], transmitting the virus to humans through contamination of food or the environment with urine, saliva, or feces [5]. Epidemiological studies indicate that Lassa fever outbreaks exhibit a marked seasonal pattern, typically peaking between December and April, and there is increasing evidence of transboundary spread [6]. Therefore, Lassa fever is considered a continuously evolving public health threat in West Africa and a growing global health concern.

Currently, laboratory diagnostic methods for LASV primarily include virus isolation, RT-PCR, ELISA, and emerging rapid antigen-based platforms, each with distinct advantages and limitations [7]. Virus isolation is considered the “gold standard” but requires biosafety level 4 (BSL-4) laboratories, limiting its routine applicability [8]. Although RT-PCR offers high sensitivity, its performance is affected by the genetic diversity of LASV, primer specificity, and equipment stability, resulting in variable sensitivity across viral lineages [9]. ELISA is useful for detecting IgM and IgG antibodies and for seroepidemiological studies, but its sensitivity during early infection is limited and the assay is time-consuming [10]. Rapid diagnostic tests (RDTs/LFIA) provide operational simplicity but lack sufficient sensitivity and specificity [11]. Consequently, the development of a rapid, sensitive, and specific antigen detection method is urgently required for effective Lassa fever surveillance and control [12].

AlphaLISA is a homogeneous, no-wash immunoassay technology based on energy transfer between donor and acceptor beads. It offers advantages such as the elimination of washing steps, short assay time, high sensitivity, and compatibility with automated and high-throughput workflows [13]. Through proximity-based energy transfer, AlphaLISA achieves efficient signal amplification and can complete detection within approximately 30 min, with higher sensitivity than that of conventional ELISA. The method has demonstrated excellent performance in detecting various viral pathogens—for example, achieving 97.9% concordance with RT-qPCR results in rotavirus detection and a detection limit of 0.024 ng/mL in Nipah virus detection [14], representing over a 40-fold improvement in sensitivity compared with ELISA [15]. Furthermore, AlphaLISA exhibits good matrix tolerance in complex samples such as serum, biological fluids, and food matrices [16], underscoring its potential for rapid on-site screening and emergency diagnostics.

In recent years, single B-cell cloning technology has emerged as an efficient approach for generating high-affinity monoclonal antibodies. This method enables the direct isolation of antigen-specific B cells from immunized animals or convalescent individuals, followed by amplification of naturally paired heavy and light chain genes through single-cell RT-PCR [17]. Compared with the traditional hybridoma technique, the single B-cell approach offers higher screening efficiency, shorter development time, and eliminates the limitations of cell fusion failure and random clonal selection, allowing rapid acquisition of antibodies with superior affinity and specificity. When combined with the AlphaLISA platform, this strategy enables a streamlined, integrated workflow from antibody discovery to assay development, significantly accelerating the establishment of sensitive immunodetection systems [18].

Based on these technologies, this study targeted the LASV-G and generated high-affinity rabbit monoclonal antibodies using single B-cell cloning. An AlphaLISA-based homogeneous immunoassay system was subsequently established. By screening optimal antibody pairs and optimizing assay parameters, a rapid, sensitive, and specific antigen detection method was developed. The established AlphaLISA system provides a novel and effective approach for the early diagnosis and epidemiological surveillance of Lassa virus and lays the groundwork for the rapid detection of other highly pathogenic viruses.

## 2. Materials and Methods

### 2.1. Materials and Instruments

The LASV-G was purchased from Sino Biological (Beijing, China). AlphaLISA acceptor beads were obtained from VDO (Suzhou, China), and donor beads were purchased from PerkinElmer (Waltham, MA, USA). 1-Ethyl-3-(3-dimethylaminopropyl) carbodiimide hydrochloride (EDC) and N-hydroxysuccinimide (NHS) were obtained from Sigma-Aldrich (St. Louis, MO, USA). Morpholineethanesulfonic acid (MES) buffer, bovine serum albumin (BSA), Tween-20, and Proclin-300 were analytical-grade reagents. The viral antigens used in this study included Ebola virus nucleoprotein (NP; Creative Diagnostics, Shirley, NY, USA), yellow fever virus NS1 protein (Creative Diagnostics, Shirley, NY, USA), dengue virus NS1 protein and Zika virus NS1 protein (Fitzgerald, Xi’an, China), as well as influenza type B and type A antigens (Sinovac Biotech, Beijing, China). Instruments included an LSRFortessa flow cytometer (Becton Dickinson, Franklin Lakes, NJ, USA), an ÄKTA protein purification system (Cytiva, Marlborough, MA, USA), a SpectraMax i3 multimode fluorescence reader (Molecular Devices, San Jose, CA, USA), an incubator (Sanyo, Osaka, Japan), and a pH meter (Mettler Toledo, Columbus, OH, USA).

### 2.2. Preparation and Purification of Monoclonal Antibodies

Rabbit monoclonal antibodies were generated following immunization with recombinant LASV-G antigen synthesized and purified by a commercial service provider (Sino Biological). The LASV-G antigen corresponds to a truncated protein (residues 53–426) derived from the glycoprotein precursor of Lassa virus strain Nig08-A47 (GenBank: ADU56630.1), a member of genetic lineage II. This construct encompasses GP1 and a portion of GP2, and was expressed and purified using a transient HEK293 expression system. New Zealand White rabbits were immunized subcutaneously with recombinant LASV-G emulsified in complete Freund’s adjuvant for the primary immunization, followed by multiple booster immunizations with antigen formulated in incomplete Freund’s adjuvant at defined intervals.

Peripheral blood mononuclear cells (PBMCs) were collected from immunized rabbits, and LASV-G-specific B cells were isolated by fluorescence-activated cell sorting (FACS). Immunoglobulin heavy and light chain genes were amplified from single B cells by reverse transcription PCR and cloned into eukaryotic expression vectors, followed by transient expression in HEK293 cells. The expressed antibodies were purified using Protein A affinity chromatography, and the buffer was exchanged to PBS. Antibody purity was verified by SDS-PAGE under reducing conditions containing 5% β-mercaptoethanol. Antibody concentrations were determined using a NanoDrop 2000 spectrophotometer (Thermo Fisher Scientific, Waltham, MA, USA), and purified antibodies were stored at 4 °C until use.

### 2.3. Labeling of Acceptor Beads

Acceptor beads were covalently coupled to antibodies using an EDC/NHS activation method. A total of 250 μg of acceptor beads (5 mg/mL) was resuspended in 0.05 M MES buffer (pH 6.0), followed by the addition of 1 μL each of EDC and NHS solutions (10 mg/mL). The mixture was incubated at room temperature for 30 min. After centrifugation and removal of the supernatant, the beads were resuspended in MES buffer (pH 7.0), and 25 μg of antibody was added. The reaction was performed at 37 °C with gentle rotation for 2 h. Subsequently, 25 μL of 2% BSA solution was added to block residual active sites, and incubation continued for an additional 2 h at 37 °C. The reaction products were washed twice with 0.05 M Tris buffer (pH 8.0) by centrifugation (16,000× *g*, 15 min, 4 °C) and finally resuspended in storage buffer (PBS containing 0.2% Tween-20, 0.2% BSA, and 0.05% Proclin-300). The labeled beads were stored at 4 °C until use.

### 2.4. Biotin Labeling

For biotin labeling, 100 μg of antibody was reacted with biotin (EZ-Link Sulfo-NHS-LC-LC-Biotin, Thermo Fisher Scientific, Waltham, MA, USA) in a final volume of 100 μL and incubated with gentle rotation for 1 h. The reaction mixture was purified using Zeba™ Spin desalting columns (7K MWCO, Thermo Fisher Scientific, Waltham, MA, USA). The post-reaction antibody was added to the column, centrifuged (1000× *g*, 1 min), and the supernatant was collected as the biotinylated antibody. The concentration of all biotinylated antibodies was adjusted to 0.5 mg/mL and then employed directly in antibody pairing screening and detection optimization experiments in the AlphaLISA system.

### 2.5. Antibody Pair Screening and Ratio Optimization

Acceptor bead-labeled antibodies were diluted at a ratio of 1:160, and the biotinylated antibodies were diluted at 1:6000. The reaction was performed in an opaque 96-well microplate by sequentially adding 20 μL of the acceptor bead–biotin mixture and 10 μL of LASV-G antigen at concentrations of 100, 1, and 0 ng/mL. The mixture was incubated at 37 °C for 15 min, followed by the addition of 20 μL of donor beads (diluted 1:250) and further incubation at 37 °C for 10 min. The final reaction volume was 50 μL. Signals were measured using a SpectraMax i3 multimode fluorescence reader. Antibody pairs yielding the highest signals were selected for further optimization. Biotinylated antibody ratios ranging from 1:4000 to 1:8000 were tested against 100 ng/mL antigen in order to determine the signal-to-noise ratio, and the optimal ratio was selected for subsequent system optimization.

### 2.6. Determination of Detection Limit and Assessment of Matrix Interference

To evaluate the assay sensitivity and matrix tolerance, standard curves were established in both buffer solution and diluted pooled healthy human serum using the AlphaLISA system. The LASV-G antigen was serially diluted twofold from 100 ng/mL to 0.00078 ng/mL, with each concentration tested in triplicate. The LOD was calculated as the mean blank signal plus three times the standard deviation (mean_blank + 3SD). Signal curve fitting was performed using a four-parameter logistic (4PL) regression model with Sigmaplot 15.0 software.

### 2.7. Evaluation of Assay Reproducibility and Specificity

Reproducibility was assessed at three distinct antigen concentrations (10, 1, and 0.1 ng/mL) with 12 intra-assay replicates for each concentration. The CV was calculated, and a CV of less than 10% was considered indicative of acceptable assay reproducibility. To ensure assay specificity, potential cross-reactivity was evaluated against a panel of antigens from other viral pathogens associated with acute febrile illness and commonly considered in the differential diagnosis. The panel included the NP protein of Ebola virus, the NS1 proteins of Dengue virus, yellow fever virus, and Zika virus, as well as whole virus preparations of influenza A (subtypes H3N2 and H1N1pdm09) and influenza B (Yamagata lineage and Victoria lineage). All assays were performed under conditions identical to those used for LASV-G detection. Signals below the cutoff value indicated no cross-reactivity.

### 2.8. Comparative ELISA Assay

To compare the performance of the AlphaLISA assay with that of conventional methods, an indirect sandwich ELISA was conducted in parallel for comparison. A 96-well high-binding capacity microplate was coated with capture antibody (0.5 μg/mL, 100 μL per well) and incubated overnight at 4 °C. After washing three times, wells were blocked with 3% BSA at 37 °C for 2 h. Diluted LASV-G antigen (100–0.0125 ng/mL, 100 μL per well) was added, followed by incubation at 37 °C for 1 h and three additional washes. Horseradish peroxidase (HRP)-conjugated detection antibody (1:10,000 dilution, 100 μL per well) was then added and incubated for 30 min at room temperature. After washing, TMB substrate was added for 10 min for color development, and the reaction was terminated with 2 M H_2_SO_4_. Absorbance was measured at 450 nm (OD_450_). Standard curve fitting and LOD calculations were performed as described for the AlphaLISA assay, using a four-parameter logistic (4PL) model in Sigmaplot 15.0.

### 2.9. Statistical Analysis

Experimental data were analyzed using GraphPad Prism 9.0 and Sigmaplot 15.0 software. Standard curves were fitted using a four-parameter logistic (4PL) model. The signal-to-noise ratio was calculated as the ratio of the detected signal to the blank signal.

## 3. Results

### 3.1. Evaluation of Antibody Purification Quality

**Figure 1:**
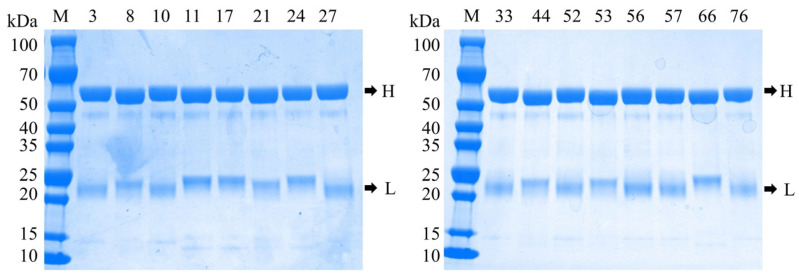
SDS-PAGE analysis of purified anti-LASV-G monoclonal antibodies. H: heavy chains; L: light chains.

The anti-LASV-G monoclonal antibodies purified by Protein A affinity chromatography were evaluated for quality using SDS-PAGE under reducing conditions. SDS-PAGE analysis revealed that all 16 antibody clones exhibited two distinct bands at approximately 50 kDa and 25 kDa, corresponding to the heavy and light chains of IgG, respectively (Figure 1). No evident degradation products or nonspecific protein bands were observed, indicating high antibody purity and structural integrity. The heterogeneity in the molecular weight of the light chains is primarily attributable to the diversity in variable region sequences driven by differences in epitope specificity. Rabbit monoclonal antibodies generate a highly diverse antibody repertoire through somatic hypermutation and gene conversion mechanisms, resulting in more pronounced inter-clonal variability in variable region sequences than typically observed in murine monoclonal antibodies. Furthermore, the sequence length and compositional diversity at the amino terminus, along with structural features at the carboxyl terminus of rabbit light chains, contribute substantially to the observed variability in electrophoretic mobility.

### 3.2. Screening of Antibody Pairs for the AlphaLISA Detection System

**Figure 2:**
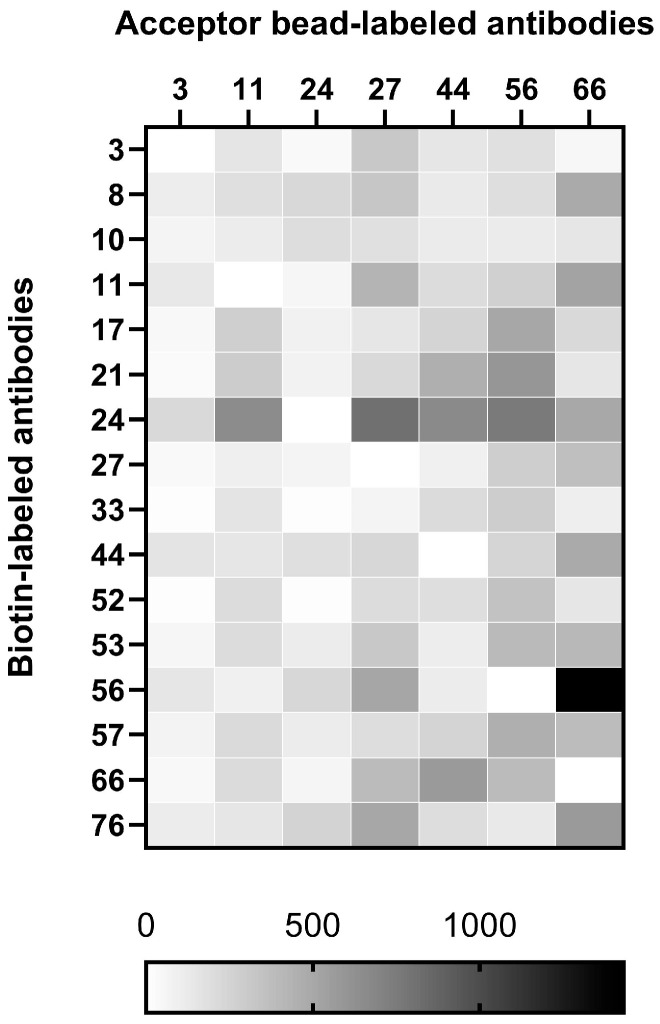
Heatmap of AlphaLISA antibody pair screening.

To establish a high-performance AlphaLISA detection system, sixteen purified anti-LASV-G monoclonal antibodies were subjected to both acceptor bead conjugation and biotin labeling for heterologous pairing in a sandwich assay format. During the acceptor bead conjugation process, several antibodies exhibited poor coupling efficiency with the bead surface, resulting in visible aggregation that compromised assay stability. After evaluation, six antibodies exhibiting stable conjugation to the acceptor beads were selected. These antibodies served as the capture antibodies (to be conjugated to beads) and were tested in all possible heterologous pairings with the sixteen biotin-labeled detection antibodies. Based on comparative analysis of the signal-to-noise ratio among different antibody pair combinations, five antibody pairs—11–24, 27–24, 44–24, 56–24, and 66–56—were identified as the top-performing combinations (Figure 2).

### 3.3. Optimization of the Biotinylation Ratio

**Figure 3:**
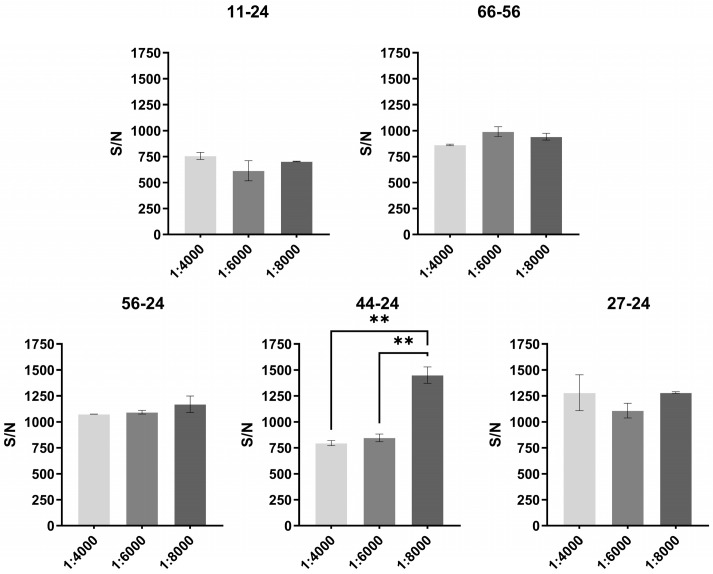
Signal-to-noise performance of five antibody pairs in the AlphaLISA assay at different biotinylation ratios. ** *p* < 0.01.

To determine the optimal biotinylation ratio for the AlphaLISA detection system, five selected antibody pairs were tested at different levels of biotin labeling. Using 100 ng/mL of LASV-G antigen as the detection target, the signal-to-noise performance was compared across a dilution range from 1:4000 to 1:8000. A higher signal-to-noise ratio was observed at a biotinylation ratio of 1:8000 (Figure 3). Considering both detection performance and system consistency, a biotinylation ratio of 1:8000 was selected as the standard condition for subsequent experiments.

### 3.4. Sensitivity

**Figure 4:**
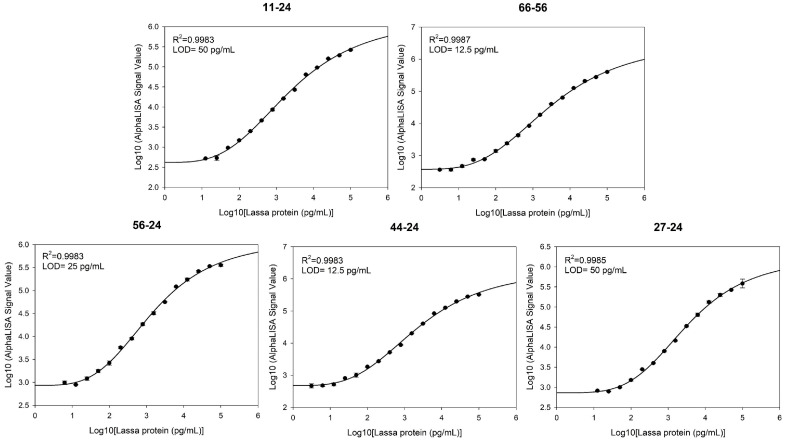
Standard curves and LOD for five antibody pairs in the AlphaLISA assay.

To evaluate the detection sensitivity of the five antibody pairs, serially diluted LASV-G antigens (0.00078–100 ng/mL) were analyzed using the AlphaLISA assay. Standard curves were fitted using a four-parameter logistic (4PL) regression model, and the LOD was calculated as the mean blank signal plus three standard deviations (mean + 3SD). The LODs of the antibody pairs were as follows: 11–24, 50 pg/mL; 27–24, 50 pg/mL; 44–24, 12.5 pg/mL; 56–24, 25 pg/mL; and 66–56, 12.5 pg/mL (Figure 4).

### 3.5. Evaluation of Serum Matrix Tolerance

**Figure 5:**
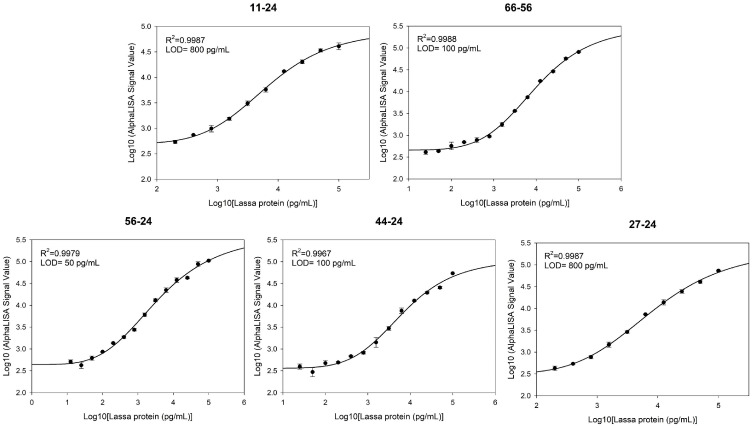
AlphaLISA detection performance of five antibody pairs in pooled healthy human serum matrix.

To evaluate the applicability and anti-interference performance of the AlphaLISA detection system in complex biological samples, pooled healthy human serum was used to assess matrix tolerance. At high antigen concentrations, the signal intensity in the serum matrix was slightly reduced compared to that in the buffer. However, the overall dose-response characteristics and linear correlation remained largely comparable (R^2^ > 0.99). Notably, the assay sensitivity varied considerably among different antibody pairs in the serum matrix. The LOD of the five antibody pairs in the serum matrix were determined as follows: 11–24, 800 pg/mL; 27–24, 800 pg/mL; 44–24, 100 pg/mL; 56–24, 50 pg/mL; and 66–56, 100 pg/mL (Figure 5). Based on their performance in the serum matrix, the 56–24 antibody pair, which demonstrated the highest sensitivity, was selected as the optimal combination for the AlphaLISA-based Lassa virus detection assay in this study.

### 3.6. Repeatability of the Optimal Antibody Pair (56–24)

The optimal antibody pair 56–24 was selected as the core component of the AlphaLISA detection system. Repeatability was assessed at three LASV-G antigen concentrations (10, 1, and 0.1 ng/mL) with twelve replicates (*n* = 12) for each concentration. The CVs at all concentration levels were below 8%, indicating good stability and repeatability of the assay under varying antigen concentrations (Table 1). These findings further confirmed the reliability of the AlphaLISA detection system and demonstrated that the assay constructed with the 56–24 antibody pair enables consistent detection of LASV-G antigen.

### 3.7. Specificity of the Optimal Antibody Pair (56–24)

**Figure 6:**
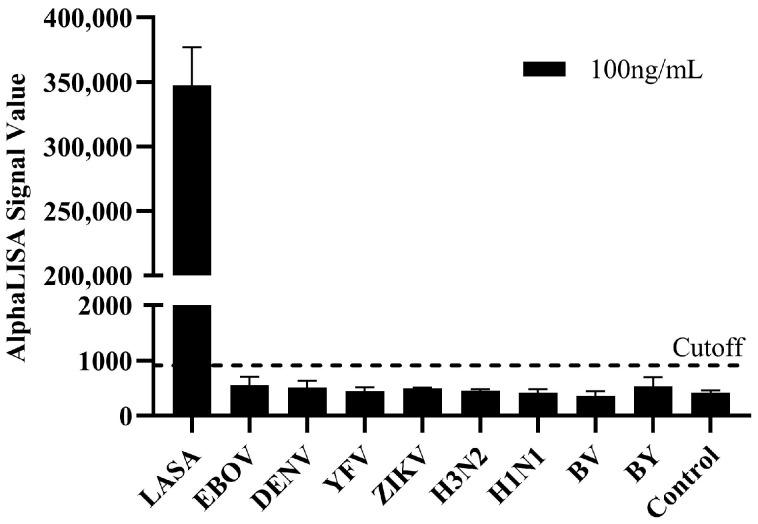
Specificity of the AlphaLISA detection system.

To verify the specificity of the established AlphaLISA detection system, cross-reactivity tests were conducted using 100 ng/mL of viral antigens or inactivated viruses from other viral pathogens associated with acute febrile illness and commonly considered in the differential diagnosis. This panel included Ebola virus nucleoprotein (NP), dengue virus NS1, yellow fever virus NS1, Zika virus NS1, and the influenza viruses: influenza A virus (subtypes H3N2 and H1N1pdm09) and influenza B virus (Yamagata and Victoria lineages). The experimental conditions were identical to those used for LASV-G antigen detection. The LASV-G antigen generated a significant specific signal, while all other tested antigens produced signal intensities below the cutoff value, indicating the absence of cross-reactivity (Figure 6). These findings demonstrate that the AlphaLISA detection system possesses excellent specificity and can accurately distinguish Lassa virus from other viral pathogens that cause clinically similar febrile illnesses.

### 3.8. Comparative Evaluation Between AlphaLISA and ELISA

**Figure 7:**
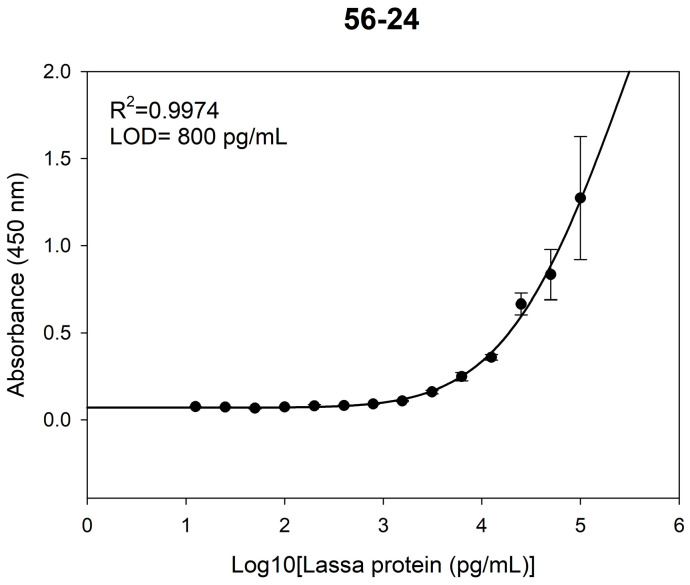
Evaluation of ELISA Detection Performance.

To further compare the performance of the two detection systems, parallel detection and validation of the same samples were conducted using the conventional ELISA. The LOD of ELISA for Lassa virus glycoprotein was 0.8 ng/mL (Figure 7). At low concentrations (<0.8 ng/mL), the signal intensity was comparable to that of the negative control and below the detection threshold (mean_blank + 3SD), indicating limited detection capability at low antigen concentrations. Consistent with the AlphaLISA reproducibility testing, the ELISA was assessed at the same three concentrations (10, 1, and 0.1 ng/mL). While CVs at 10 and 1 ng/mL were satisfactory (<8%), the CV at 0.1 ng/mL was higher (11.54%), which is attributed to the concentration approaching the assay’s detection limit, leading to greater data variability (Table 2). This observation underscores ELISA’s relative limitation in low-concentration detection.

## 4. Discussion

Lassa fever, an acute hemorrhagic disease caused by Lassa virus, remains a significant and ongoing threat to global public health. The disease is endemic in West Africa and has, in recent years, caused several cross-continental outbreaks. Currently, there are no approved vaccines, and clinical management relies primarily on antiviral drugs such as ribavirin combined with supportive care. Since therapeutic efficacy is closely linked to the timing of intervention, rapid and accurate diagnostics are crucial for effective disease control and prevention [19]. Against this backdrop, targeting the LASV-G—a core antigen for immune recognition—we combined single-B-cell-cloning-derived high-affinity antibodies with AlphaLISA technology to develop a rapid, wash-free diagnostic platform.

The LASV-G was selected as the target for developing specific antibodies and constructing a highly sensitive and specific immunoassay due to its role as a core antigen for immune recognition and diagnostic detection. While nucleoprotein is the most abundant viral protein in LASV-infected cells and virions, and thus a commonly used target in immunoassays, the glycoprotein complex (GPC) is the only surface-exposed glycoprotein and the sole mediator of host cell entry [20,21]. Therefore, in the context of our AlphaLISA platform, targeting GPC offers the distinct advantage of detecting intact, replication-competent virions, which provides a more functionally relevant indicator of active infection compared to detecting the highly abundant but internal nucleoprotein. GPC exhibits favorable stability and immunoreactivity [22]. Structural studies have confirmed that appropriately stabilized glycoprotein complexes can maintain their native conformation and induce robust immune responses [23]. Furthermore, cross-lineage comparative analyses indicate that the spatial structure of the glycoprotein complex is highly conserved among different LASV strains [24], making it a reliable and stable diagnostic antigen.

To obtain high-quality monoclonal antibodies (mAbs) targeting this complex, we employed single-B-cell cloning technology. While traditional hybridoma technology has long been the standard method for mAb generation, it involves animal immunization and cell fusion, resulting in a lengthy process with relatively limited efficiency. In contrast, single-B-cell cloning technology enables the direct isolation of antigen-specific B cells from immunized animals and allows for the recovery of natively paired heavy- and light-chain sequences within weeks [25]. Published studies have also reported that integrating this technique with microfluidics and high-throughput screening significantly enhances antibody discovery efficiency [26]. Utilizing this approach, we successfully generated high-affinity rabbit mAbs, providing the core molecular reagents for subsequent assay development.

Among existing diagnostic methods for LASV, reverse transcription–polymerase chain reaction (RT-PCR) is considered the gold standard due to its high analytical sensitivity and specificity. However, it has drawbacks, including high cost, operational complexity, and long turnaround times [27]. Therefore, immunoassays serve as a vital complementary tool for rapid screening. Although the traditional ELISA offers good specificity, its requirement for multiple washing steps and prolonged incubation limits signal accumulation at low antigen concentrations [28]. Our parallel validation experiments confirmed this limitation, as the ELISA signal became indistinguishable from the background noise at antigen concentrations below 0.8 ng/mL, highlighting its inadequacy for trace antigen detection.

To overcome these technical constraints, we developed a detection platform based on AlphaLISA technology. This homogeneous, wash-free method utilizes energy transfer between donor and acceptor beads to achieve signal amplification, thereby enhancing both sensitivity and speed. Previous studies have confirmed a high concordance between AlphaLISA and RT-PCR results, with a significant reduction in processing time [14]. In our optimized assay, the selected antibody pair (56–24) achieved a limit of detection as low as 25 pg/mL for the target antigen, representing an approximately 30-fold improvement in sensitivity over ELISA. The assay also demonstrated excellent repeatability in both buffer and serum matrices, with coefficients of variation below 8%. These results confirm that this AlphaLISA platform enables the rapid, highly sensitive, and reliable detection of LASV-G antigen. While other emerging technologies, such as single-particle interferometric reflectance imaging sensor (SP-IRIS), have demonstrated ultra-high sensitivity and multiplexing capabilities in research settings [29], their reliance on sophisticated optical equipment and chip fabrication limits their practicality for point-of-care or field applications. In contrast, AlphaLISA combines high sensitivity with operational simplicity, making it more suitable for clinical and near-patient testing scenarios.

The detection system developed in this study has certain limitations, including a modest reduction in signal intensity in serum matrices and the use of recombinant GPC antigen. Further optimization and validation using well-characterized clinical specimens representing diverse LASV lineages are therefore required. In addition, potential cross-reactivity with lymphocytic choriomeningitis virus (LCMV), Tacaribe virus, and other arenaviruses remains to be systematically excluded. In the context of the recently published international Target Product Profiles (TPPs) for Lassa virus diagnostics (May 2025) [30], the AlphaLISA-based assay meets several core attributes of the TPP2 profile for laboratory-based confirmatory tests, such as high analytical sensitivity, rapid turnaround time, and wash-free operation, but it does not address the operational simplicity and cost requirements defined for point-of-care use (TPP1). Accordingly, this work should be regarded as a laboratory-based methodological validation that provides a sensitive and adaptable platform for LASV antigen detection and potentially for other highly pathogenic viruses in reference laboratory settings.

## 5. Conclusions

In this study, multiple high-affinity rabbit monoclonal antibodies targeting the LASV-G were successfully generated, and the optimal antibody pair (56–24) was identified. Based on this pair, a wash-free and homogeneous AlphaLISA detection system was developed, enabling rapid and highly sensitive detection of LASV-G. The established assay exhibited excellent linearity and reproducibility in both buffer and serum matrices, with a minimum detection limit of 0.025 ng/mL—approximately 30-fold higher sensitivity than conventional ELISA. These findings demonstrate that the AlphaLISA platform possesses high sensitivity, rapid response, and strong matrix tolerance, providing an efficient and practical approach for early diagnosis and epidemiological surveillance of Lassa virus, as well as a methodological reference for rapid on-site detection of other highly pathogenic viruses.

## Figures and Tables

**Table 1:** Repeatability analysis of the AlphaLISA detection system based on the 56–24 antibody pair.

Concentration (ng/mL)	Mean (*n* = 12)	SD	CV (%)
10	217,508	17,120	7.87
1.0	34,268	2441	7.13
0.1	3931	268	6.84

**Table 2:** Repeatability analysis of the ELISA detection system.

Concentration (ng/mL)	Mean (*n* = 12)	SD	CV (%)
10	0.564	0.035	6.16
1.0	0.178	0.014	7.70
0.1	0.131	0.015	11.54

## Data Availability

The data that support the findings of this study are available from the corresponding author upon reasonable request.

## Abbreviations

The following abbreviations are used in this manuscript:

AlphaLISA	Amplified Luminescent Proximity Homogeneous Assay
BSA	Bovine serum albumin
BSL-4	Biosafety level 4
CV	Coefficient of variation
EDC	1-Ethyl-3-(3-dimethylaminopropyl) carbodiimide
ELISA	Enzyme-linked immunosorbent assay
FACS	Fluorescence-activated cell sorting
GPC	Glycoprotein complex
HRP	Horseradish peroxidase
LASV	Lassa virus
LASV-G	Lassa virus glycoprotein complex
LF	Lassa fever
LOD	Limit of detection
MES	Morpholineethanesulfonic acid
mAb	Monoclonal antibody
NP	Nucleoprotein
NS1	Non-structural protein 1
PBMC	Peripheral blood mononuclear cell
PBS	Phosphate-buffered saline
RDT	Rapid diagnostic test
RT-PCR	Reverse transcription polymerase chain reaction
SD	Standard deviation
TMB	3,3′,5,5′-Tetramethylbenzidine
TPP	Target Product Profile
